# P-320. National Estimates of Rates and Resistance Trends of Enterococci Cases in the United States, 2012-2022

**DOI:** 10.1093/ofid/ofae631.523

**Published:** 2025-01-29

**Authors:** Alex Maillis, Natalie McCarthy, Joseph D Lutgring, Sujan Reddy, Hannah Wolford, James Baggs

**Affiliations:** CDC, Acworth, Georgia; CDC, Acworth, Georgia; Division of Healthcare Quality Promotion, Centers for Disease Control and Prevention, Atlanta, GA; CDC, Acworth, Georgia; CDC, Acworth, Georgia; CDC, Acworth, Georgia

## Abstract

**Background:**

Enterococci are common causes of both community-onset (CO) and hospital-onset (HO) infections; vancomycin-resistant enterococci (VRE) are a serious threat. We sought to describe national estimates of *Enterococcus faecalis* and *E. faecium* rates and the proportion of cases that were vancomycin-resistant from 2012-2022

**Figure:**
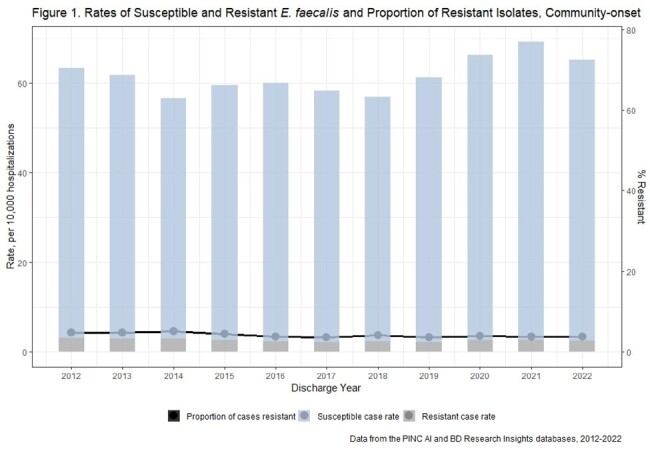

**Methods:**

We identified a cohort of patients from the PINC AI and BD Research Insights databases with a clinical culture yielding an E. faecalis or E. faecium isolate with accompanying antimicrobial susceptibility testing (AST). Cultures obtained from rectal, perirectal or nasal swabs were excluded. E. faecalis or faecium isolates resistant to vancomycin were considered VRE. Isolates from patients with no culture yielding the same species and vancomycin AST phenotype in the previous 14 days were counted as an incident case. CO cultures were obtained ≤ day 3 of hospitalization; HO cultures were obtained ≥ day 4. We used a raking-procedure to determine weights for extrapolating the number of discharges included in our sample to match the national distribution of discharges, stratified by bed size, U.S. census division, urban/rural designation, and teaching status, for U.S. hospitals included in the American Hospital Association survey. Weighted rates were calculated over time as cases per 10,000 hospitalizations. The proportion of VRE (%R) was calculated as the rate of VRE cases divided by the rate of all cases of that species with an AST result.

**Figure:**
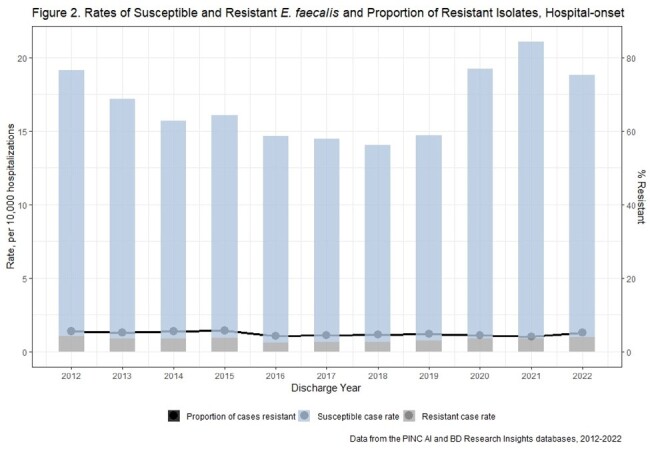

**Results:**

There were 810 unique acute care hospitals from 2012-2022. Weighted estimates resulted in 124,570 E. faecalis and 516,724 E. faecium incident cases nationally. Rates of CO and HO E. faecalis and E. faecium declined from 2012-2018 and increased from 2018-2021 (Figures 1-4). E. faecalis %R for vancomycin remained stable over the study period (CO: 4-5%, HO: 4-6%), while E. faecium %R declined over the study period from 67% to 57% among CO cases, and from 78% to 70% among HO cases.

**Figure:**
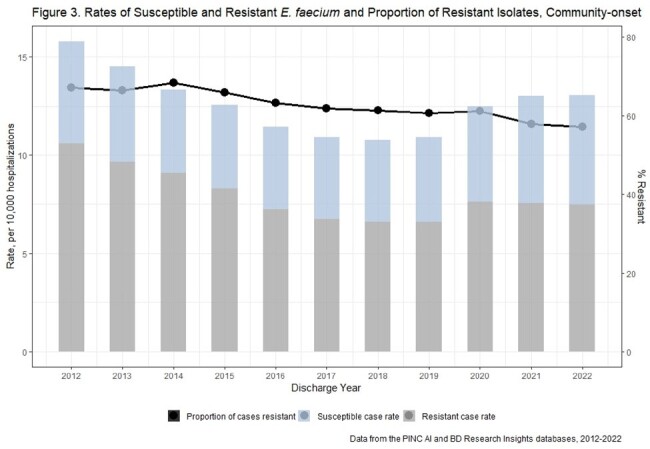

**Conclusion:**

Enterococci and VRE rates have increased recently while the proportion of resistant isolates has been stable for E. faecalis and falling for E. faecium. This suggests rate increases may be due to factors affecting all enterococci (e.g., host susceptibility or pathogen factors) rather than VRE specific factors (e.g., vancomycin exposure).

**Figure:**
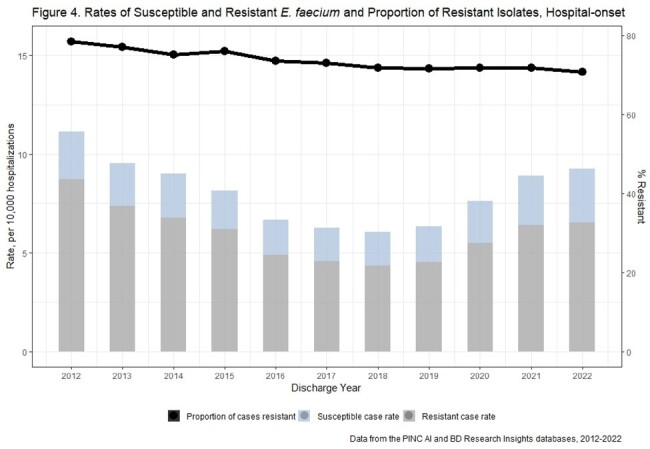

**Disclosures:**

**All Authors**: No reported disclosures

